# Surgical Instruments

**Published:** 1876-03

**Authors:** 


### SURGICAL INSTRUMENTS

Are quite the rage now-a-days. Men and women, as "flat as a flounder," patronize abdominal supporters, when the great mischief is, they havn't anything to support.

Deaf women, the dumb ones having all died off before the flood, are provided with patent "Auricles," which stick out on each side of the head, like two great rams' horns, all regardless of the fact, whether there is any hearing to be aided or not. Then there are *shoulder braces* and *back straps*, *respirators*, *inhalers*, *et id omne*, *ad infinitum;* so that there is scarcely a member of the human body that is not provided with an "*aid;*" the stomach has a million, among the worst, are stinking German gin and British beer, made out of worse than bilge-water. Now, any intelligent physician knows that the vast mass of persons who patronize these great variety of supporters, need aids of a very different kind. The best respirator in the world is, to *shut your mouth and go ahead;* the most efficient "shoulder brace," is to hold up your head and march on; while the most valuable *general* "supporter," and the only one needed in nine cases out of ten, is to make the patient go to work and compel him to live on his daily earnings.

